# Molecular and Pathogenetic Aspects of Tumor Budding in Colorectal Cancer

**DOI:** 10.3389/fmed.2015.00011

**Published:** 2015-03-10

**Authors:** Heather Dawson, Alessandro Lugli

**Affiliations:** ^1^Clinical Pathology Division, Institute of Pathology, University of Bern, Bern, Switzerland; ^2^Translational Research Unit, Institute of Pathology, University of Bern, Bern, Switzerland

**Keywords:** tumor budding, colorectal cancer, immunohistochemistry, EMT, tumor microenvironment, immunohistochemistry

## Abstract

In recent years, tumor budding in colorectal cancer has gained much attention as an indicator of lymph node metastasis, distant metastatic disease, local recurrence, worse overall and disease-free survival, and as an independent prognostic factor. Tumor buds, defined as the presence of single tumor cells or small clusters of up to five tumor cells at the peritumoral invasive front (peritumoral buds) or within the main tumor body (intratumoral buds), are thought to represent the morphological correlate of cancer cells having undergone epithelial–mesenchymal transition (EMT), an important mechanism for the progression of epithelial cancers. In contrast to their undisputed prognostic power and potential to influence clinical management, our current understanding of the biological background of tumor buds is less established. Most studies examining tumor buds have attempted to recapitulate findings of mechanistic EMT studies using immunohistochemical markers. The aim of this review is to provide a comprehensive summary of studies examining protein expression profiles of tumor buds and to illustrate the molecular pathways and crosstalk involved in their formation and maintenance.

## Introduction

The hallmark of malignant disease, namely the ability of a tumor to disseminate and colonize distant sites, requires an arsenal of cellular characteristics. In colorectal cancer and a growing number of solid tumors, epithelial–mesenchymal transition (EMT) is proposed as a fundamental mechanism for epithelial cells to acquire such a “malignant” phenotype (1, 2). Tumor buds, defined as the presence of single tumor cells or small groups of up to five tumor cells at the invasive tumor front or within the main tumor body [termed intratumoral buds (3)] are thought to be the histomorphological correlate of cells to undergo EMT in colorectal cancer. Indeed, high-grade tumor budding is strongly and independently associated with many adverse features such as vascular invasion, lymph node, and distant metastases and is detrimental to overall and disease-free survival (4–12).

**Figure 1:**
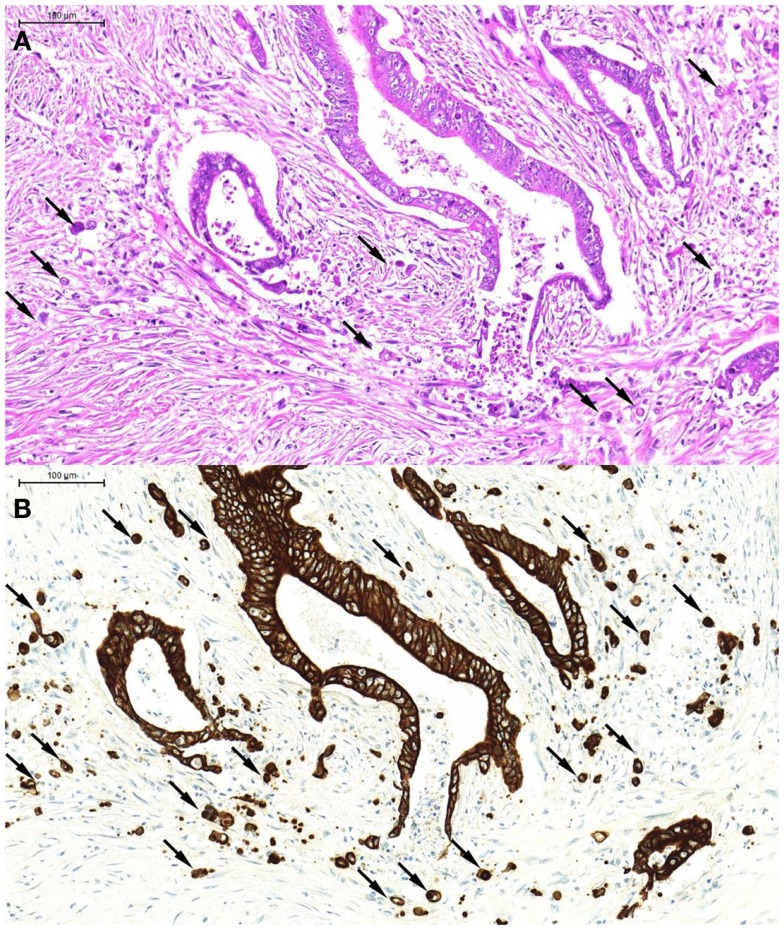
**An example of tumor budding (single tumor cells and small clusters of up to five tumor cells detached from the main tumor body)**. These can be seen on H&E [**(A)**, arrows] but are far more readily recognized on the pancytokeratin immunostain of the corresponding tumor area [**(B)**, arrows].

From a morphological point of view, tumor buds tend to appear more atypical than their counterparts in the main tumor body [hence the previous term “tumor dedifferentiation” (13)] Tumor buds may be difficult to detect on H&E stained slides, as they are, per definition, single tumor cells or small clusters of tumor cells that have broken off from the main tumor body and “blend” into the tumor microenvironment, often obscured by peritumoral inflammatory reaction. At high power, it may be difficult to distinguish tumor buds from reactive stromal cells, which may also appear large and atypical. Pancytokeratin immunostains are of great help in the accurate identification of tumor buds (Figures 1A,B), and have been demonstrated to significantly improve interobserver agreement in tumor bud assessments (14).

As tumor buds are visualized in a histological “snapshot,” studies using immunohistochemistry have been pivotal in improving our understanding of tumor buds and their protein expression profiles (15). A distinct heterogeneity in immunohistochemical expression profiles among different tumor compartments (center vs. invasive front and tumor buds) has contributed to our appreciation of the consequences of EMT (15).

## Molecular Background of Tumor-Budding Phenotypes

It is well-recognized that “colorectal cancer” encompasses fundamentally different molecular phenotypes following various pathways of carcinogenesis (16). As a consequence, colorectal cancers arising from different pathways differ in terms of biological behavior, histomorphological features, and protein expression (17, 18). One of the major and most well-studied pathways involves mutation of the APC gene, activating the WNT/wingless signaling pathway. Its major downstream effectors, β-catenin, and E-cadherin, are considered integral components of EMT (1). Therefore, it is not surprising that high-grade tumor budding is strongly associated with tumors arising from the tumors with mutation of the APC gene (19). In contrast, tumors with microsatellite instability, another well-studied pathway of colorectal carcinogenesis, are inversely correlated with tumor budding (19, 20). To date, only few studies have systematically assessed differences in tumor bud expression profiles taking into account the molecular background of tumors. In MMR-deficient tumors, reduced β-catenin expression in tumor buds was demonstrated in comparison to MMR-proficient tumors (21), leading to the speculation that mechanisms other than only Wnt signaling may lead to the formation of tumor buds in MMR-deficient cancers. Also, buds arising in MMR-deficient tumors may represent a less aggressive budding phenotype, highlighted by reduced expression of the cell locomotion protein laminin5γ2 in buds and in line with the generally milder clinical course of these tumors (21). On the other hand, adenocarcinomas with “serrated” morphology, which did not display histological features associated with microsatellite instability, were shown to have increased expression of laminin5γ2 and decreased expression of nuclear β-catenin and E-cadherin in tumor-budding cells compared to matched “conventional” adenocarcinomas (22). Unfortunately, the classification of these tumors was made based on morphology alone, hence their true molecular background remains presumptive [serrated morphology without features of MSI-high tumors being most suggestive of BRAF-mutated, CIMP-high, MMR-proficient tumors, which are known to behave aggressively (23, 24)].

## Wnt Signaling

Activation of WNT signaling leads to stabilization of membranous/cytoplamasmic β-catenin and its translocation to the nucleus. Located at the cell membrane, β-catenin complexes with E-cadherin and is crucial for maintaining cell–cell adhesion and epithelial cell polarity (24). However, mutations in the APC gene lead to nuclear translocation of β-catenin, where it binds to members of the Tcf/LEF family, and functions as an oncogenic transcription factor. Therefore, preservation of membranous E-cadherin and β-catenin are indicative of an epithelial phenotype, whereas loss of E-Cadherin and nuclear expression of β-catenin are considered hallmarks of EMT. Due to their well-established role in EMT, β-catenin and its transcriptional targets represent the most extensively studied group of proteins in tumor buds. Increased nuclear expression of β-catenin in tumor buds in comparison to the main tumor body has been demonstrated in several studies (21, 22, 25–29) as has loss of E-Cadherin (4, 28, 30, 31) (summarized in Table 1). However, canonical Wnt signaling and β-catenin alone appear not to be the sole driving force behind tumor budding, as nuclear β-catenin at the invasive tumor front did not necessarily predict budding (32, 33) and although up to 90% of all colorectal cancers have dysregulation of Wnt signaling and 60% harbor APC mutations (34), high-grade budding is only seen in a proportion of these (around 40%), depending on case mix and evaluation methods (9, 14, 35–37).

**Table 1:** **Summary of studies examining tumor buds by immunohistochemistry**.

Biological role	Reference	Markers and methods	Cohort	Budding systematically assessed? Scoring method (reference)	Results/relevance
Wnt signaling	Gosens (25)	EpCAM: three different antibodies (Ber-EP4, 311-1K1 and a polyclonal antibody), double staining for β-catenin and Ep-CAM. mRNA *in situ* hybridization of Ep-CAM, WTS	133 rectal cancers (Dutch RT + TME trial), Stage II–IV	Yes (Ueno) (9)	Tumor buds showed lack of membranous and increased cytoplasmic Ep-CAM staining and nuclear expression of β-catenin. Reduced Ep-CAM staining at the invasive margins correlated with tumor-budding, grade, and increased risk of LR
Wnt signaling	Brabletz (27)	β-catenin, WTS	44 Stage I–III CRC	No	Expression of nuclear β-catenin in 54% of all cases. Strong nuclear staining predominantly at tumor front (80–100%) with strongest staining in tumor buds. Tumor center often without nuclear staining but with retained membranous staining
Wnt signaling	El-Bahrawy (28)	E-Cadherin, α-, β-, and γ-catenin (each immunohistochemistry and mRNA), WTS	30 Dukes A-C CRC	No	Cytoplasmic accumulation of E-cadherin and catenins in over 80% of cases. Increased staining of β-catenin toward tumor front
Wnt signaling	Lauscher (29)	Pontin, β-catenin, WTS. Pontin western blot on six cases	34 CRC Stage I–IV	No	Cytoplasmic pontin expression in all cases, additional nuclear positivity in 50% of cases. Nuclear pontin correlated with nuclear β-catenin in all cases. Nuclear pontin staining stronger at invasive margin and tumor buds in comparison to tumor center (41.2 and 37.9% of cases). Sample size insufficient for significant correlation to stage
Wnt signaling	Garcia-Solano (22)	β-catenin, e-cadherin, p-cadherin, laminin5γ2, SMAD4, WTS	20 SAC (defined by histomorphologic criteria, no features of MSI-high tumors) with stage matched 20 CAC	Yes (Ueno)	Increased expression of laminin5γ2, decreased expression of nuclear β-catenin and membranous e-cadherin in tumor buds of SAC in comparison to CAC
Wnt signaling	Shinto (21)	laminin5γ2, β-catenin (assessed in tumor buds), MUC2, MUC5AC (assessed on entire tumor), WTS. Laminin5γ2 promoter methylation	80 CRC with high-grade budding: 9 sporadic MMR-deficient, 7 Lynch MMR-deficient and 64 sporadic MMR-proficient, Stage n/a	Yes (Ueno)	3/9 sporadic MMRd laminin5γ2 compared to 46/64 sporadic MMRp (*p* 0.05) and 2/7 Lynch (*p* = 0.03). Nuclear β-catenin more frequent in MMRp than MMRd cancers (*p* 0.01). No difference in methylation among subsets but correlation between methylation and negative laminin5γ2
Cell differentiation cell cycle	Harbaum (30)	CK7, CK20, E-cadherin, MUC2, and MIB1. CK7: 370 cancers on multi-punch TMA, CK7 positive cases re-evaluated on WTS with all markers	370 CRC Stage I–IV	Yes (Ueno)	32 cases positive for CK7. CK7 positivity prevailed in tumor buds, these cells were positive for CK20 and negative for E-Cadherin, MUC2 and MIB1 on serial sections. Raises the notion of “EET” (epithelial–epithelial transition)
Wnt signaling	Brabletz (31)	CK18, β-catenin, e-cadherin, Ki-67, WTS	72 CRC Stage n/a	No	Nuclear β catenin in tumor buds accompanied by reduced E-cadherin and Ki-67 reactivity, inverse immunoprofile in main tumor and metastases
Wnt signaling	Horkko (32)	Tumor-budding margin on all cases, β-catenin (108 cases), MNF116 (53 cases to assess separately for budding), WTS	466 CRC Dukes A-D	Yes (Ueno)	Nuclear β catenin increased at invasive front and in tumor buds, but no correlation between expression presence/absence of budding
Wnt signaling	Guzinska-Ustymowicz (38)	MMP-9 and cathepsin B, WTS	55 pT3 G2 CRC	Yes (Morodomi) (37)	Expression of MMP-9 and Cathepsin B associated with lymph node involvement (*p* < 0.01)
Wnt signaling	Rubio (39)	MNF116, Ki-67, laminin5	6 CRC (preliminary report), Stage n/a	Hotspot on HE	Mean positivity of buds in comparative fields: MNF 116: 86.2, Ki-67: 9.7, laminin5: 9.3
Wnt signaling	Gavert (42)	β-catenin, L1, ADAM10, WTS	25 CRC, Stage n/a	No	L1 not detected in main tumor body, but at invasive front and tumor buds, co-localization with ADAM10, and nuclear β-catenin
Wnt signaling	Gavert (52)	NFκB, L1, ezrin, WTS	25 CRC, Stage n/a	No	Tumor buds co-express ezrin, nuclear NfKb and L1, central tumor regions with relative lack immunoreactivity. Together with functional data supports hypothesis that L1-mediated activation of NFkB signaling is a major route of CRC tumor progression
CSC	Hostettler (49)	CK22, CD133, CD166, CD24, CD44s, CD90, EpCAM, ALDH1, ABCG5, evaluation within tumor buds on WTS	101 cases with densest budding out of cohort with 300 CRC patients, Stage n/a	Yes (Ueno)	CD90, CD44s, and CD133 infrequent in buds (<5%). ALDH1, CD24 and CD166 in 16.5, 16.2, and 34%. ABCG5 and EpCAM in 35 and 69% of cases. EpCAM and ABCG5 in buds significantly associated with worse prognosis, especially in node-negative patients with ABCG5 positive buds
CSC	Kleist (56)	Lgr5, WTS	89 cases Stage I–IV, additional distant metastases from 31 patients	Yes (Prall) (36)	12.9% of cases had Lgr5 positive buds, distant metastases from these cases had 6- to 11.5-fold higher expression rates
Cell cycle	Dawson (59)	Ki-67 (WTS), Caspase3, M30Cytodeath (multi-punch TMA)	188 Stage I–IV CRC	Yes (Karamitopoulou) (35)	Ki-67 expression in 0.3% of buds, in 35% tumor center (*p* 0.0001). Caspase-3 comparatively lower in tumor buds than other compartments (*p* 0.0001). Rare cases with Ki-67 and caspase3 immunoreactivity associated with poorer prognosis
RAS/RAF	Koelzer (67)	RKIP, NFkB, E-Cadherin WTS RKIP, matched NFκB, and E-Cadherin on multi-punch TMA	178 Stage I–IV CRC	Yes (Karamitopoulou)	0.9% of tumor buds positive for RKIP, but expression in main tumor body rather than buds predictive for metastatic disease, vascular invasion, budding, and invasive tumor border configuration. RKIP expression correlated with NFkB expression
RAS/MAPK	Dawson (68)	TrkB, multi-punch TMA	211 Stage I–IV CRC	Yes (Karamitopoulou)	Trkb(m) overexpressed in buds in comparison to main tumor body (*p* < 0.0001) and associated with KRAS mutation. High expression of membranous Trkb-independent adverse prognostic factor. Inverse correlations between expression profile of Trkb(m) and Ki-67 as well as Caspase-3 (53)
Cytokine signaling	Akishima-Fukusawa (71)	CXCL12, WTS	165 Stage II–III CRC	Yes (Ueno)	CXCL12-positive budding divided into high- and low-grade, staining in the tumor divided into high and low expression. Patients with high-grade CXCL12 budding and high CXCL12 expression had shorter survival than patients with low-grade CXCL12 budding and low CXCL12 expression. CXCL12 expression in buds independent adverse prognostic factor in multivariate analysis irrespective of budding grade
Wnt signaling, cell differentiation	Brabletz (76)	β.catenin, Cdx2, laminin5γ2 WTS, additional to cell culture experiments and immunofluorescence	45 CRC cases, Stage n/a	No	Cdx2 expression was lost in tumor buds but re-expressed in metastases, cell culture experiments demonstrate transient transcriptional down-regulation of Cdx2 triggered by collagen type I
Stromal cell interaction	Galvan (79)	TWIST1 and TWIST2 immunohistochemistry on 2 cohorts: cohort 1 (multi-punch TMA) + promoter methylation. Cohort 2: TMA from pre-operative biopsies (prognostic effects). Immunohistochemistry for both markers and promoter methylation in six cell lines. LCM in one tumor-budding high and one tumor-budding low case	Cohort 1: 185 Stage I–IV CRC, Cohort 2: 112 Stage I–IV CRC	Yes [cohort 1: Karamitopoulou, cohort 2: Zlobec (3)]	TWIST 1 and 2 expression restricted to stromal cells. Inverse correlation between TWIST1 protein expression and methylation (Cohort 1) suggests hypermethylation as a mechanism of TWIST1 regulation. TWIST 1 and 2 protein expression significantly correlated with low- and high-grade budding phenotype. LCM of high-grade tumor-budding case with positive TWIST1/2 stroma and no methylation, inverse pattern in low-grade tumor-budding case. TWIST1 (Cohort 2) associated with adverse tumor features and independent prognostic factor.
Stromal cell interaction	Karagiannis (81)	Bone morphogenic protein antagonists HTRA3, FST and GREM1, markers assessed in tumors and cancer-associated fibroblasts, WTS	2 cohorts: 1:30 patients with 10 each no, low and high-grade budding. 2: 219 Stage II CRC	Yes (Ueno)	HTRA3 staining in the epithelial tumor component was differentially regulated between areas with and without tumor-budding, correlation between HETRA3 staining and the presence of budding and with significantly increased expression in tumor-budding cells themselves. Epithelial HTRA3 expression-independent adverse prognostic factor

*WTS, whole tissue sections; LR, local recurrence; SAC, serrated adenocarcinomas; CAC, conventional adenocarcinomas; MMRd, mismatch repair deficient; MMRp, mismatch repair proficient; LCM, laser capture microdissection*.

The functions of proteins encoded by WNT target genes confer characteristics of a malignant mesenchymal phenotype. Proteins involved in the degradation of the extracellular matrix, such as MMP-9 and Cathepsin B have been shown to be overexpressed in buds (38). Several studies have demonstrated expression of the cell locomotion protein laminin5γ2 in buds (21, 22, 39, 40).

Other cell adhesion proteins such as EpCAM have been implicated in the budding process, with loss of membranous expression identified in tumor buds (25). EpCAM is activated by proteolysis by tumor-necrosis factor alpha (TNF α) converting enzyme, resulting in release of EpICD into the cytoplasm, which becomes part of the h-catenin and LEF transcriptional complex (41). The neuronal cell adhesion molecule L1 has also been identified as a β-catenin target gene and is preferentially expressed in tumor buds where it is co-regulated with ADAM10, a metalloprotease involved in cleaving and shedding L1s extracellular domain (42). L1 has recently been demonstrated to induce NFκB signaling in colorectal cancer cells (52), NFκB being implicated in EMT (53). These studies demonstrate the degree of crosstalk between Wnt signaling and EMT.

Modulators of Wnt signaling have also been detected in tumor buds, such as the AAA+ protein family member pontin (29), which has been implicated in enhancing the effect of Wnt signaling by binding to the β-catenin/LEF complex.

## Tumor Buds, EMT, and “Stemness”

The stem-cell concept is centered on the notion that tumor progression is driven by a primarily undifferentiated population of tumor-initiating cancer cells. After initially being described in acute myeloid cancers, cancer stem cells (CSCs) have been identified in a myriad of solid tumors including colorectal cancers. CSCs display aggressive features such as increased invasiveness, chemoresistance, and the ability to mediate angiogenesis and resist apoptosis, with the ability to re-differentiate at metastatic sites (54). It would therefore stand to reason that tumor buds may represent a population of migrating CSCs (55). Indeed, there is increasing evidence linking CSCs to EMT. For instance, forced expression of the EMT transcription factor snail in CRC cell lines leads to increased expression of the putative stem-cell markers CD133 and CD44 (43). Alleged stem-cell markers in colorectal cancer include EpCAM (alongside its role as a cell adhesion molecule), CD133, CD44, ABCG5, CD90, CD24, CD166, LGR5 (a Wnt pathway target), and ALDH1 (49). Several studies have examined the immunohistochemical expression of stem-cell markers in different compartments of colorectal cancer. CD133 has been reported to be preferentially expressed at the invasive tumor front but not within tumor buds themselves (44). Hostettler et al. (49) found expression of CD133, 166, CD44, and CD90 to be a rare event in tumor buds, but cytoplasmic EpCAM and ABGC5 were frequently expressed in tumor-budding cells. Both of these markers were demonstrated to have a negative effect on survival, and expression of ABCG5 in buds was associated with worse prognosis in node-negative colorectal cancer patients. A study examining the expression of Lgr5 found a small subset of buds to be positive for this putative stem-cell maker but 6- to 11.5-fold higher expression rates in distant metastases were detected (56). Taken together, the above results support the notion that expression of stem-cell markers appears to be heterogeneous among buds and that only small populations of tumor cells (low-frequency subclones) may be perpetrators of metastatic disease.

## Cell Cycle-Related Proteins

There is accumulating evidence indicating that the driving force of colorectal cancer progression may not be attributable to tumor cell proliferation alone. Generally, it is thought that EMT-derived tumor cells are hypo-proliferative, underlining the significance of aggressive cellular machinery to exert their malignant properties (57). The cell cycle regulators cyclin D1 and p16 are Wnt signaling targets and their activation is a suggested mechanism of EMT-induced growth arrest. Under normal circumstances, nuclear p16 is a direct inhibitor of cyclin D1, arresting the cell cycle. However, located in the cytoplasm, p16 is thought to bind with CDK4, blocking its transport to the nucleus. CDK4 is required for cyclin D1 activation. Therefore, in the absence of CDK4, cyclin D1 forms an inactive complex with CDK2, accounting for the apparently paradoxical co-upregulation of p16 and cyclin D1 (58). Indeed, tumor buds have been demonstrated to show cytoplasmic expression of p16 (19, 57). As a consequence, several studies have demonstrated the hypo-proliferative nature of the invasive front and tumor buds themselves using Ki-67 immunohistochemistry (30, 39, 45, 59).

As the hypo-proliferative nature of tumor buds is gaining recognition, it may be speculated that in order to survive migration through stroma, tumor buds must confer of essential survival mechanisms. In fact, single epithelial cells detached from the extracellular matrix are programed to undergo a certain form of cell death termed anoikis (60). In addition to their hypo-proliferative state, tumor buds have been demonstrated to be anti-apoptotic by their relative lack of immunoreactivity for caspase 3 (59), suggesting that tumor buds are able to resist anoikis.

## EMT-Inducing Pathways Involving RAS/RAF and RAS/MAPK Signaling

Cancer cells frequently exploit growth factor signaling from the surrounding microenvironment (such as insulin growth factor, hepatocyte growth factor, epidermal growth factor, or placental-derived growth factor) to drive tumor progression (61–63). Well-studied downstream pathways include PI3K-, NFκB-, Snail, and RAS–RAF–ERK–ZEB1 (Figure 1). For instance, as Snail controls ZEB1, a transcriptional repressor of E-cadherin (64); it is not surprising that genes involved in growth factor signaling induce EMT.

The tumor suppressor gene RKIP has been linked to EMT on several levels, for one as inhibitor of the Ras–Raf–MEK–ERK signaling cascade at the level of Raf (65). In addition, RKIP modulates other signaling pathways including NFκB–Snail (46, 66). Several studies have demonstrated differential expression of RKIP in zones of colorectal cancer, with gradual loss of expression toward the tumor front (33, 67) and ability of RKIP expression to predict high-grade budding. RKIP was only rarely detected in tumor buds and in line with mechanistic EMT studies, loss of RKIP correlated with E-Cadherin negativity and nuclear translocation of NFκB. However, the prognostic significance of RKIP appears to be restricted to its expression in the tumor center, suggesting that other mechanisms may become increasingly important in the development of tumor-budding cells (67).

The neurotrophic tyrosine kinase receptor TrkB has been linked to EMT via RAS/MAPK-dependent Twist–Snail signaling and has been demonstrated to be a potent and specific suppressor of anoikis (47), which is supported by its overexpression in tumor buds (68). Additionally, KRAS-mutated colorectal cancers also overexpress TrkB, in concordance with the known dependency on MAPK signaling on TrkB-induced EMT.

## CXCL12 (sdf-1)/CXCR4 Pathway

Chemokines, integral for cell migration and trafficking, are widely expressed by cells of the lymphatic and hemopoietic systems. The chemokine CXCL12 binds to its receptor CXCL4, activating subsequent intracellular pathways involved in chemotaxis, cell survival, and gene transcription (69). As CXCR4 is expressed in cells in multiple organs including lymph nodes, lungs, and liver, epithelial tumor cells may take advantage of the principle of homing mechanisms to direct the metastasis of CXCL12-positive tumor cells to CXCR4 positive organs (70). CXCL12 can also stimulate the formation of capillary structures (48). CXCL12 expression in tumor buds was found to be correlated with liver metastases and was an independent prognostic marker (71).

## Markers of Intestinal Differentiation

The homeobox transcription factor Cdx2 encodes a transcription factor specific to intestinal differentiation, which is essential for development and homeostasis of gut epithelium (72). Recent evidence also suggests that Cdx2 may play a substantial role in Wnt signaling as a tumor suppressor gene and therefore inhibit EMT. For instance, Cdx2 has been found to bind β-catenin, thus disrupting the β-catenin/TCF complex (73). Also, Cdx2 may inhibit the transcriptional activity of β-catenin through interaction with the protocadherin Mucdhl (74). Finally, Cdx2 enhances the function of E-cadherin by trafficking it to the cell membrane, thus restoring cell adhesion (75). As dedifferentiated cancer cells, it is not surprising that tumor buds lack expression of Cdx2 (76). However, the fact that most colorectal cancers that diffusely express Cdx2 also do so in their metastases (as the marker may be used diagnostically for cancers of unknown primary as a marker of intestinal differentiation) supports the notion of tumor redifferentiation and reversibility of EMT at metastatic sites.

Few studies have examined the expression of other markers of epithelial differentiation in tumor buds. For instance, Harbaum et al (30) demonstrated absence of the intestinal-type mucin Muc2 and overexpression of cytokeratin 7, a simple intermediate keratin filament, at the invasive front and strikingly in tumor buds. This finding is intriguing since expression of cytokeratin 7 is relatively infrequent in primary colorectal carcinoma (77), and because intermediate filaments are traditionally known to support cell–cell or cell–matrix adhesions in epithelial cells. However, recent evidence suggests that keratin filaments may contribute to a higher degree of cellular plasticity than originally assumed (78) and it may be postulated that surrounding biochemical and mechanical stimulation in the tumor microenvironment could influence the cytoskeletal protein composition.

## Stromal–Epithelial Interaction in the Tumor Microenvironment

It has also been postulated that signals derived from surrounding mesenchymal cells in the tumor microenvironment may play a significant role in facilitating a pro-budding phenotype (50, 79). For instance, immunohistochemical expression of TWIST1 and TWIST2, known activators of EMT, was significantly positively correlated with a tumor-budding phenotype (both low-grade and high-grade budding), yet their expression was virtually restricted to stromal cells in the tumor microenvironment. Moreover, in high-grade budding cancers an inverse correlation between TWIST1 methylation and stromal protein expression was observed, suggesting hypermethylation as a mechanism of TWIST1 regulation (79). TWIST1 has previously been demonstrated to be expressed in neoplastic stromal cells. These cells were shown to be neoplastic, demonstrating the same neoplastic aberrations as the tumor itself, indicating that EMT had indeed taken place with cells having acquired a fully mesenchymal phenotype (80).

The interplay between epithelial and stromal components has also been underlined by studies examining bone morphogenetic protein (BMP) antagonists (51, 81), hypothesizing that CRC cells in the tumor microenvironment can only flourish in a milieu devoid of BMP signaling, this was characterized immunohistochemically by a shift in HTRA3 expression patterns (decreased stromal staining and increased epithelial staining).

## Conclusion

Tumor budding is thought to represent the morphological correlate of EMT in colorectal cancers and has been strongly linked to adverse clinicopathological features and poor overall and disease-free patient survival. These consistent associations indicate that tumor budding has a strong value as a prognostic indicator, and it has been proposed that budding should be an integrated category in pathology reports (82).

**Figure 2:**
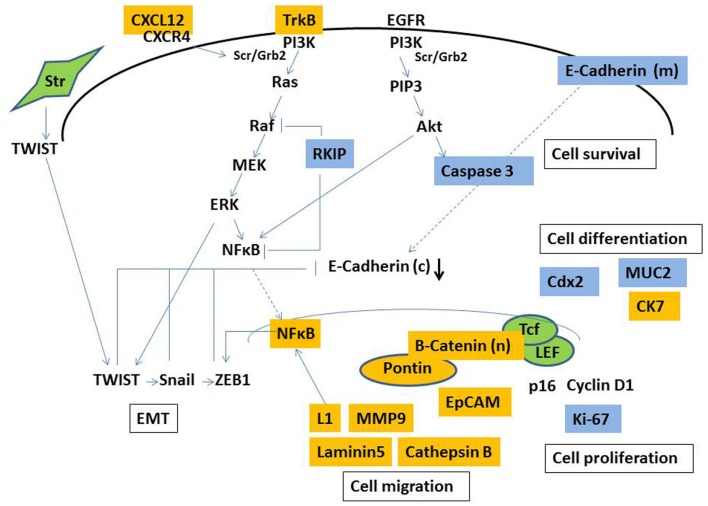
**Simplified illustration of molecular pathways involved in the formation of tumor budding**. Markers demonstrated to be overexpressed (yellow) and underexpressed (blue) in tumor buds by immunohistochemistry. Str, stromal cell, (c), cytoplasmic, (m) membranous, (n) nuclear.

In an attempt to contribute to our understanding of tumor buds, previous studies have mainly immunohistochemistry to discriminate properties unique to tumor-budding cells. The main reason for this is that immunohistochemistry enables the actual identification of tumor buds for evaluation. To our knowledge, there is currently no method of extracting tumor buds from fresh tumor tissue, presenting huge hurdles for molecular studies specifically geared at tumor buds. Therefore, our understanding of the biology of tumor buds is essentially restricted to protein expresssion profiles (as visualized in Figure 2), and a few studies, which have used mRNA *in situ* hybridization. Immunohistochemistry as a semi-quantitative method may be especially prone to subjectivity, and staining intensity greatly depends on laboratory methods (83). Such issues may contribute to difficulties in reproducibility and the consistency of results. Not all proteins differentially expressed in tumor buds appear to have significant prognostic relevance, which may be at least in part explained by the timing of certain events in the process of carcinogenesis and the accumulation of different simultaneous molecular occurrences, of which our knowledge is limited.

Therefore, although the molecular background of colorectal cancers appears to play an important role in budding, much remains to be investigated in terms of genetic profiles of tumor buds and how various molecular pathways are taken advantage of by these cells to maintain their malignant phenotype and drive tumor progression. Novel areas of interest include the interaction of tumor buds with cancer-associated fibroblasts and inflammatory cells in the tumor microenvironment (84) and the evasion of anoikis. Taken together, and based on growing evidence that tumor buds may be targetable structures (15), our understanding of these mechanisms will be crucial for the development of future therapies aimed at the destruction of tumor buds.

## Conflict of Interest Statement

The authors declare that the research was conducted in the absence of any commercial or financial relationships that could be construed as a potential conflict of interest.
